# N_2_-to-NH_3_ conversion by excess electrons trapped in point vacancies on 5*f*-element dioxide surfaces

**DOI:** 10.3389/fchem.2022.1051496

**Published:** 2023-01-05

**Authors:** Gaoxue Wang, Enrique R. Batista, Ping Yang

**Affiliations:** Theoretical Division, Los Alamos National Laboratory, Los Alamos, NM, United States

**Keywords:** N_2_-to-NH_3_ conversion, excess electrons, point vacancies, actinide dioxide surfaces, DFT density functional theory

## Abstract

Ammonia (NH_3_) is one of the basic chemicals in artificial fertilizers and a promising carbon-free energy storage carrier. Its industrial synthesis is typically realized *via* the Haber−Bosch process using traditional iron-based catalysts. Developing advanced catalysts that can reduce the N_2_ activation barrier and make NH_3_ synthesis more efficient is a long-term goal in the field. Most heterogeneous catalysts for N_2_-to-NH_3_ conversion are multicomponent systems with singly dispersed metal clusters on supporting materials to activate N_2_ and H_2_ molecules. Herein, we report single-component heterogeneous catalysts based on 5*f* actinide dioxide surfaces (ThO_2_ and UO_2_) with oxygen vacancies for N_2_-to-NH_3_ conversion. The reaction cycle we propose is enabled by a dual-site mechanism, where N_2_ and H_2_ can be activated at different vacancy sites on the same surface; NH_3_ is subsequently formed by H^−^ migration on the surface *via* associative pathways. Oxygen vacancies recover to their initial states after the release of two molecules of NH_3_, making it possible for the catalytic cycle to continue. Our work demonstrates the catalytic activities of oxygen vacancies on 5*f* actinide dioxide surfaces for N_2_ activation, which may inspire the search for highly efficient, single-component catalysts that are easy to synthesize and control for NH_3_ conversion.

## Introduction

The industrial synthesis of ammonia (NH_3_) from gaseous N_2_ and H_2_ molecules is typically realized *via* the Haber−Bosch process, one of the most significant catalytic reactions discovered in the early 20th century (Grunze, 1982; Logadottir and Nørskov, 2003; Schlögl, 2015). Over 200 million tons of NH_3_ is produced annually, 80% of which is used in artificial fertilizers for agriculture to feed Earth’s growing population (Giddey et al., 2017). Since NH_3_ contains 17.6 wt% hydrogen, it is also considered to be a promising carbon-free energy storage intermediate that can be more easily stored and transported than gaseous H_2_. The industrial Haber−Bosch process, activated by Fe- and Ru-based catalysts, must be conducted under harsh conditions (high pressure: 150–300 atm and high temperature: 300–600 °C) to overcome kinetic limitations and to achieve a sufficient yield of NH_3_ (Hammer and Nørskov, 2000; Galloway et al., 2008; Cherkasov et al., 2015). This is because of the inertness of the triply bonded, non-polar N_2_ molecule, which exhibits a large bond energy of 941 kJ mol^−1^, negative electron affinity, and high ionization energy (Gambarotta and Scott, 2004; Pool et al., 2004; Walter, 2016). For Fe- and Ru-based catalysts, NH_3_ is synthesized *via* a dissociative mechanism, where N_2_ first dissociates at active sites, and the dissociated *N is subsequently hydrogenated step-by-step to produce NH_3_ (Bozso et al., 1977; Ertl et al., 1982; Logadottir and Nørskov, 2003; Reuter et al., 2017). The dissociation of N_2_ into two *N is generally accepted as the rate-determining step for the synthesis of NH_3_ (Temkin and Pyzhev, 1940; Stoltze and Nørskov, 1985; Ertl, 2008; Kitano et al., 2015). The development of new catalysts to reduce the N_2_ activation barrier, enabling the synthesis of NH_3_ with a high yield under mild conditions, is highly desired in the field.

During the search for new catalysts, scientists have been inspired by the biological N_2_ fixation process using nitrogenase enzymes under ambient temperature and pressure conditions. These enzymes contain metal clusters, such as the Fe−Mo cofactor, providing active sites for the binding and reduction of N_2_ (Rodriguez et al., 2011; Foster et al., 2018). New approaches to synthesize NH_3_ using electrocatalysis, photocatalysis, and plasma catalysis have been developed (Patil et al., 2015; Li et al., 2017; Guo et al., 2018; Hong et al., 2018; Yang et al., 2018; Li et al., 2020). Several metal cluster and molecular catalysts have been used in these reactions (Anderson et al., 2013; Kerpal et al., 2013; Shima et al., 2013; Grubel et al., 2014; Hölscher and Leitner, 2015; McWilliams and Holland, 2015; Tyler, 2015; Araake et al., 2017; Ling et al., 2019). Although very promising, NH_3_ yields obtained using these alternative methods are very low, and the processes are still in their infancy, failing to meet the requirements for practical use (Li et al., 2020). Recently, enlightened by the booming use of single-atom catalysts or single-cluster catalysts (Geng et al., 2018; Ling et al., 2018; Wang et al., 2018; Hannagan et al., 2020; Mitchell and Pérez-Ramírez, 2020), several multicomponent heterogeneous catalysts, composed of atomically dispersed metal atoms or clusters on supporting substrates for N_2_ reduction, have been proposed (Choi et al., 2018; Niu et al., 2020). Li et al. proposed the use of singly dispersed Fe_3_ or Rh_1_Co_3_ supported by Al_2_O_3_(010) or CoO(011) for NH_3_ synthesis (Liu et al., 2018; Ma et al., 2018; Shi et al., 2022). Different from the dissociative mechanism of the Haber−Bosch process, the synthesis of NH_3_ using Fe_3_ or Rh_1_Co_3_ occurs *via* an associative mechanism—mimicking the mechanism in nitrogenase-based synthesis (Liu et al., 2018; Ma et al., 2018). Recent work has demonstrated that the Ni-loaded LaN surface enables stable and highly efficient NH_3_ synthesis (Ye et al., 2020a). It was found that NH_3_ was synthesized through a dual-site mechanism, with N_2_ activation by surface N vacancies and H_2_ activation by Ni clusters. Since singly dispersed metal clusters are highly unstable and prone to agglomeration due to their high surface energy (Yang et al., 2013), precise control of the morphology, size, and stability of these multicomponent heterogeneous catalysts remains challenging (Speck et al., 2021).

Significant technological advances will be gained by identifying a highly efficient, single-component system for N_2_-to-NH_3_ conversion that is easy to synthesize and manufacture. Inspired by our recent observation of a unique phenomenon in early 5*f*-element materials (Wang et al., 2019), we hypothesize that AnO_2_ (An = Th and U) surfaces are promising candidates for such catalysts. In that work, we reported that excess electrons due to atomic oxygen vacancies on the surfaces of 5*f*-element materials can spontaneously induce catalytic water splitting (Wang et al., 2019). Our computational studies found that excess electrons remained at the vacancy site on ThO_2_ and UO_2_ instead of moving to the metal centers. These excess electrons at the vacancy site are extremely reactive and can spontaneously induce chemical reactions even with inert molecules. Hence, it is intriguing to investigate if these excess electrons can activate N_2_ molecules and simplify the N_2_-to-NH_3_ conversion by using a single-component system. If the results are positive, what is the unique role of the 5*f* orbitals in the activation of N_2_? For example, uranium exhibits a large range of oxidation states, from +II to + VI, which expands the scope of its accessible intermediates and products for chemical reactions.

Actinide-containing materials have been used in catalytic applications (Jackson and Hargreaves, 2008; Ismagilov and Lazareva, 2013; Falcone et al., 2017; Leduc et al., 2019). Uranium-containing compounds are efficient catalysts in organic syntheses (Barbier-Baudry et al., 2000) and in the activation of small molecules such as CO, H_2_O, CH_4_, and HCl (Leduc et al., 2019). This is because of the large ionic radii of actinides and the active participation of 5*f* electrons in the valence space that can lead to unique reactivity patterns and product distributions not accessible to transition metal complexes (Leduc et al., 2019). Haber first applied metallic uranium and uranium nitrides to synthesize NH_3_ in 1909, which also had to be conducted under high pressure and temperature conditions (Rossignol, 1910; Leduc et al., 2019). Recently, a number of uranium complexes that can bind to the N_2_ molecule have been reported (Evans et al., 2003; Falcone and Mazzanti, 2018; Lu et al., 2019; Jori et al., 2021; Keener et al., 2021; Xin et al., 2021); however, the full catalytic cycle of NH_3_ formation has not been realized using these molecular uranium complexes. However, actinide dioxide surfaces containing oxygen defects have never been tested for NH_3_ synthesis.

In this work, we studied the activation of N_2_ for NH_3_ synthesis using singly dispersed atomic vacancies on ThO_2_ and UO_2_ surfaces that can be easily generated by Ar ion sputtering (Senanayake et al., 2007a; Senanayake et al., 2007b). We demonstrate, using first-principle calculations, that the atomic oxygen vacancy can serve as the active site for NH_3_ synthesis from N_2_ and H_2_ gases. We show that N_2_ can be chemically adsorbed at the vacancy site. H_2_ can dissociate directly at the vacancy site, forming two H^−^ ions. The migration of H^−^ on the surface can lead to the hydrogenation of *NN and the subsequent formation of NH_3_ through an associative mechanism. Our work demonstrates that early 5*f*-element materials are promising candidates as high-efficiency single-component catalysts for N_2_-to-NH_3_ conversion.

## Methods

All calculations were performed with the use of density functional theory (DFT) and the projector augmented-wave (PAW) method (Kresse and Joubert, 1999), implemented in the Vienna *Ab initio* Simulation Package (VASP) (Kresse and Furthmüller, 1996). The generalized gradient approximation (GGA) of the Perdew–Burke–Ernzerhof (PBE) (Perdew et al., 1996) functional was used to represent the exchange−correlation interaction. Since the PBE functional does not capture the van der Waals (vdW) dispersion energy (Giovannetti et al., 2007; Sachs et al., 2011), the DFT-D3 method described by Grimme (Grimme, 2006) was included in the calculations. Plane wave basis sets with a cutoff energy of 520 eV were employed (Kresse and Joubert, 1999). The energy convergence was set to 10^–6^ eV and the residual force on each atom upon geometry optimization was below 0.01 eV/Å. The (111) surfaces of ThO_2_ and UO_2_ were represented with slab models, with a vacuum gap in the direction normal to the surface (Wang et al., 2017). The vacuum distance normal to the slab was larger than 30 Å, in order to eliminate interactions between the periodic images due to the periodic boundary conditions. Dipole correction was included to nullify the artificial field imposed on the slab by the periodic boundary conditions (Bengtsson, 1999). For UO_2_ surfaces, a Hubbard-like on-site Coulomb interaction was included, namely, the DFT + *U* method (Dudarev et al., 1998), to treat the correlated electrons. The DFT + *U* (*U* = 4.0 eV) method overcomes deficiencies of the pure LDA/GGA functionals and has achieved a wide range of success in treating the UO_2_ system (Dorado et al., 2009; Wen et al., 2013). The reciprocal space was sampled by Monkhorst−Pack *k* points in the Brillouin zone, with a grid size of 3 × 3 × 1. Spin-orbit coupling was ignored in this work since previous studies have demonstrated that it introduces only minor corrections in reaction energies of both actinide surfaces and molecular systems (Batista et al., 2004; Rak et al., 2013; Goldman and Morales, 2017; Kelley et al., 2017; Deblonde et al., 2018; McSkimming et al., 2018; Han and Kaltsoyannis, 2022). To reduce the computational cost, the 1*k* collinear antiferromagnetic (AFM) order was used for UO_2_ surfaces, and the ThO_2_ surface was non-magnetic. A recent study has shown that non-collinear magnetism is important to completely understand the electronic structure of AnO_2_ (Pegg et al., 2020). Oxygen vacancies were created by removing a top-surface oxygen atom, corresponding to a vacancy coverage of one-fourth of the monolayer (*Θ*_*v*_ = 1/4), following our previous work (Wang et al., 2019). The chemical composition at the top surface of this model is U_4_O_7_, which has been reported experimentally (UO_2−*x*_; *x* < 0.3) (Senanayake et al., 2007a; Senanayake et al., 2007b). Transition states were searched using the climbing image nudged elastic-band method (CI-NEB) (Henkelman et al., 2000).

## Results and discussion

The formal oxidation states of the actinide and oxygen atoms in ThO_2_ and UO_2_ are +4 and −2, respectively. Therefore, a neutral oxygen vacancy is expected to generate two excess electrons on the surface, which can lead to high chemical activities of the materials. In a previous study, we found that the two excess electrons remained at the vacancy site on ThO_2_ and moved to the 5*f* orbitals of Pu on the PuO_2_ surface. Whereas on the UO_2_ surface, one of the excess electrons preferred to localize on a nearby U 5*f* orbital, leaving one excess electron at the vacancy site. This behavior is due to the different reduction potentials of the materials; from +4 to +3 as a result of the contraction and energy lowering of the 5*f* orbital level going from Th to Pu. These readily available excess electrons are extremely reactive and can spontaneously induce the reduction of water and its splitting (Wang et al., 2019). In this work, we investigate the activation of N_2_ molecules by these excess electrons at the vacancy site in the first section, followed by a discussion on the activation of H_2_ molecules in the next section. We conclude with a proposed NH_3_ formation mechanism in the last section.

### N_2_ activation at the oxygen vacancy site on ThO_2_ and UO_2_ (111) surfaces

**FIGURE 1:**
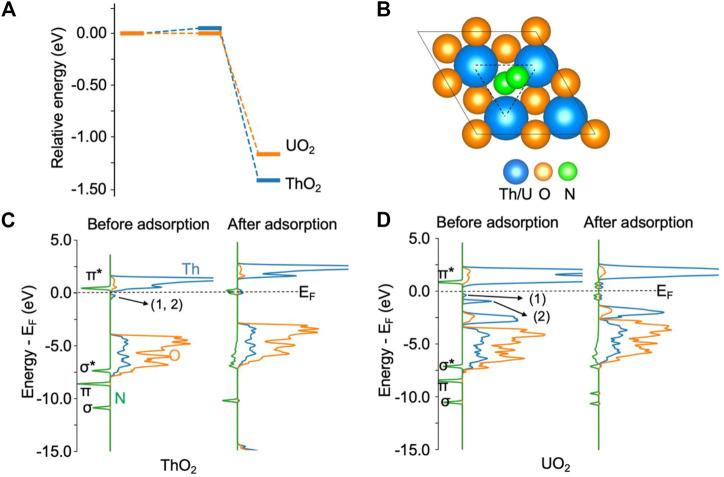
N_2_ adsorption on ThO_2_ and UO_2_ (111) surfaces containing oxygen vacancies. **(A)** Adsorption energy diagram. The total energy of the initial state is set to zero. **(B)** Illustration of structures after N_2_ adsorption. The oxygen vacancy site is highlighted by the triangle. **(C)** Density of states before and after N_2_ adsorption on the ThO_2_ surface. **(D)** Density of states before and after N_2_ adsorption on the UO_2_ surface. The two excess electrons on the ThO_2_ surface are located at the vacancy sites labeled 1 and 2 in the DOS, directly below the Fermi level (E_F_) in **(C)**. On the UO_2_ surface **(D)**, one of the excess electrons (1) is located at a vacancy site and the other (2) in uranium 5*f* orbitals.

Figure 1 shows the adsorption of N_2_ on ThO_2_ and UO_2_ (111) surfaces *via* oxygen vacancies. From the energy diagram in Figure 1A, it is seen that it is thermodynamically favorable for N_2_ to chemisorb at the vacancy site, overcoming an energy barrier smaller than 0.1 eV. The adsorption energies are −1.40 eV and −1.10 eV on ThO_2_ and UO_2_ surfaces, respectively. Upon N_2_ adsorption on the ThO_2_ surface, as shown in Figure 1B, the N−N bond length is stretched from 1.10 Å in the gas phase to 1.23 Å, indicative of a reduction in the bond order from three to two and the activation of the N≡N bond. The *NN distance and the typical N≡N, N=N, and N−N bond lengths are summarized in Supplementary Table S1. From the density of state (DOS) plots in Figure 1C, one can see that the π* orbital of the free N_2_ molecule is well-aligned with the unoccupied 5*f* orbitals of Th atoms before N_2_ adsorption, and π*−5*f* hybrid orbitals are formed near the Fermi level after N_2_ adsorption. Before N_2_ adsorption, there is a small peak in the DOS slightly below the Fermi level, corresponding to the excess electrons at the vacancy sites (Wang et al., 2019). After N_2_ adsorption, the excess electrons transfer to the π*−5*f* hybrid orbitals. Hybridization of the N_2_ σ* and *π* orbitals with the Th 2*p* orbitals is also observed from the broadening of these orbitals after N_2_ adsorption. The transfer of excess electrons and the orbital hybridization between N and Th atoms result in the stretching and weakening of the N−N bond, which is a critical step for the activation of N_2_.

On the UO_2_ surface containing an oxygen vacancy, one of the excess electrons is localized in the 5*f* orbital of the uranium atom near the vacancy. This is evidenced from the DOS before N_2_ adsorption, as shown in Figure 1D. The first small peak, labeled 1) in Figure 1D, directly below the Fermi energy, corresponds to the delocalized excess electron at the vacancy site. The second peak, labeled 2) in Figure 1D, below the Fermi energy, corresponds to the other excess electron that is localized in the uranium 5*f* orbital. The magnetic moment of this uranium atom is 2.8 *μ*_*B*_, as expected for U(III). The remaining uranium atoms maintain the U(IV) magnetic moment of 2.0 *μ*_*B*_, similar to surfaces without oxygen vacancies. After N_2_ adsorption, the magnetic moments of all uranium atoms are 2.0 *μ*_*B*_, indicating the transfer of the excess electrons to *NN. The N≡N bond is activated, as evidenced by a bond length of 1.24 Å after adsorption. Bader charge analysis shows that *NN receives 1.2 e^−^ transferred from the surface. Similarly, the calculated bond length of *NN is also in agreement with the reported bond length of 1.28 for N_2_^−^ (Thulstrup and Andersen, 1968-1987), and is close to the reported bond length of 1.25–1.28 Å for the N=N double bond (Smieja et al., 1987; Hill et al., 1991). The adsorbed *NN is spin polarized, with magnetic moments of 0.3 and 0.5 *μ*_*B*_ on each N atom, respectively.

### H_2_ activation at the oxygen vacancy site of ThO_2_ and UO_2_ (111) surfaces

**FIGURE 2:**
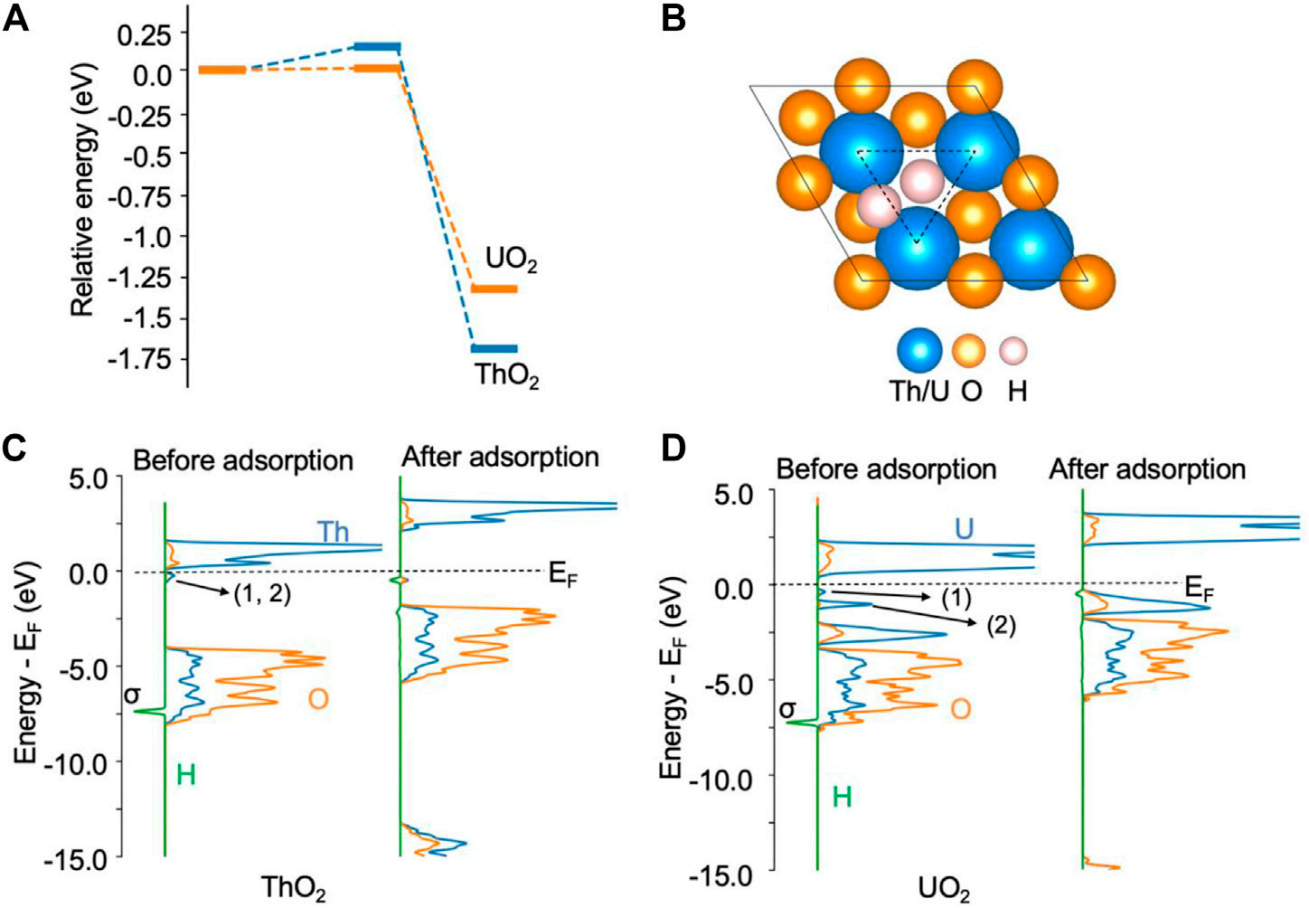
H_2_ dissociation on ThO_2_ and UO_2_ (111) surfaces containing oxygen vacancies. **(A)** Adsorption energy profile. The total energy of the initial state is set to zero. **(B)** Illustration of structures after H_2_ adsorption. The oxygen vacancy site is highlighted by the triangle. **(C)** Density of states before and after H_2_ adsorption on the ThO_2_ surface. **(D)** Density of states before and after H_2_ adsorption on the UO_2_ surface. The two excess electrons on the ThO_2_ surface are located at the vacancy sites labeled 1 and 2 in the DOS, directly below the Fermi level (E_F_) in **(C)**. On the UO_2_ surface **(D)**, one of the excess electrons (1) is located at a vacancy site and the other (2) in uranium 5*f* orbitals.

Likewise, the H_2_ molecule can be easily activated by excess electrons and can spontaneously dissociate at an oxygen vacancy site on both ThO_2_ and UO_2_ (111) surfaces. The activation barrier for this process is less than 0.2 eV, as shown in Figure 2A. On the ThO_2_ surface, after dissociation, one *H occupies a vacancy site and the other is located between two actinide atoms, as shown in Figure 2B. On most metal surfaces, *H is located at the fcc/top site, as summarized by Greeley et al. (Greeley and Mavrikakis, 2005). For the Rh_1_Co_3_/CoO(011) system, H_2_ dissociates at the Rh site and forms two Rh−H bonds (Ma et al., 2018). For the Ni-loaded LaN surface, H_2_ dissociates at the Ni cluster and forms a Ni−H bond with one Ni atom (Ye et al., 2020a). We have performed Hessian calculations for this structure and did not find imaginary vibrational frequencies, indicating that the optimization converged to a minimum energy structure. The vibrational frequency for *H at the vacancy site is in the range of 750–1243 cm^−1^, and that for *H at the bridge site of the two metal atoms is in the range of 630–907 cm^−1^. Bader charge analysis reveals that each *H receives 0.62 e^−^ from the surface, see Supplementary Table S2. We have considered the structure to contain a lattice O protonated by *H, see Supplementary Figure S1. It is found that this structure is 1.9 eV higher in energy than the structure in Figure 2B. The Bader charge of each *H is in line with the reported Bader charges in metal hydrides (Aboud and Wilcox, 2010), indicating the formation of two H^−^ ions on the surface. The formation of H^−^ has also been recently observed on the CeN surface containing nitrogen vacancies, where a Bader charge of 0.7 e^−^ was determined (Ye et al., 2020b). On the ThO_2_ surface, the H−H bond length is increased from 0.75 Å in the gas phase to 2.04 Å after dissociation. The DOS in Figure 2C shows that the *σ* orbital of H_2_ disappears after H_2_ adsorption, indicating full dissociation of H_2_. This dissociation process releases −1.68 eV of energy. A similar process is observed for H_2_ on the UO_2_ surface, where each dissociated *H has a charge of −0.56 e^−^. The formation of the two H^−^ ions is also evidenced by the disappearance of the two peaks directly below the Fermi level, as shown in Figure 2D. In contrast to state-of-the-art heterogeneous catalysts such as Ni-loaded LaN for NH_3_ synthesis (Ye et al., 2020a), where H_2_ is activated by the Ni cluster on the surfaces, and N_2_ by the N vacancy on the LaN surface, both N_2_ and H_2_ molecules can be activated by the same type of oxygen vacancy site on ThO_2_ and UO_2_. Hence, this provides the first evidence that these single-component materials containing oxygen vacancies may be able to catalyze the N_2_-to-NH_3_ conversion.

### Possible NH_3_ formation mechanisms

**FIGURE 3:**
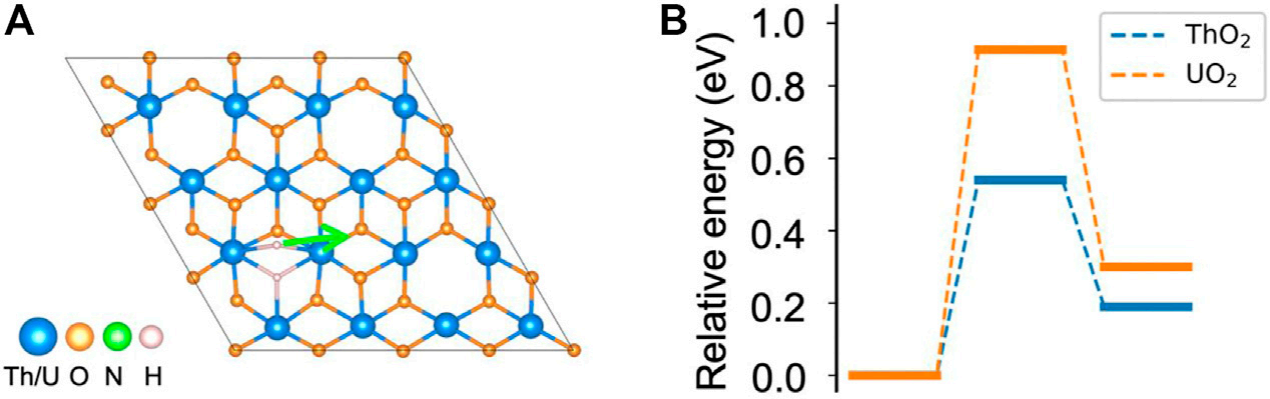
Migration of dissociated H^−^ on ThO_2_ and UO_2_ surfaces. **(A)** Migration path of H^−^ to a nearby site. **(B)** Corresponding energy diagram. The total energy of the initial state is set to zero.

As shown in the earlier sections, both N_2_ and H_2_ can be activated at the vacancy site on ThO_2_ and UO_2_ (111) surfaces. The N_2_ bond length is stretched to 1.24 Å after adsorption on the surface, indicating activation of the triple N≡N bond and formation of the N=N double bond (Smieja et al., 1987; Hill et al., 1991). This is a key step toward the breaking of the N_2_ molecule for NH_3_ synthesis. The chemically adsorbed *NN is still bound together, and direct dissociation has large energy barriers of 3.64 eV and 2.91 eV on ThO_2_ and UO_2_ surfaces, respectively, see Supplementary Figure S2. Therefore, NH_3_ synthesis is unlikely to occur *via* the dissociative mechanism on either surface. However, the H_2_ molecule can be directly dissociated to form H^−^ at another oxygen vacancy site, and the migration of H^−^ can hydrogenate *NN to form NH_3_ through associative pathways. To verify this, we calculated the migration barrier of the dissociated H^−^ on the surfaces. It is found that the dissociated H^−^ that is located between two actinide atoms has a migration barrier of 0.54 eV on ThO_2_ and 0.91 eV on the UO_2_ surface, see Figure 3. Thus, the migration of H^−^ can be driven by a relatively low temperature and can lead to the hydrogenation of *NN. We have also determined the energy for dissociated H^−^ migration to surface oxygen sites. We found that it is not energetically preferred since over1.9 eV is required for H^−^ to diffuse to the oxygen sites. It should be noted that N_2_ adsorption on the H_2_ pre-dissociated vacancy site is possible since H^−^ is reactive. However, H^−^ migration on the surface is still necessary to supply enough hydrogen for the formation of NH_3_. Based on these observations, we propose a dual-site mechanism for NH_3_ synthesis, where N_2_ and H_2_ are activated at different but neighboring oxygen vacancy sites. We limited ourselves to the thermodynamics of NH_3_ formation in this work. While the reaction barriers during the reaction cycle are important for the kinetics and NH_3_ formation rates, a large number of reaction paths need to be considered to sample possible reaction processes, which is computationally expensive at the DFT theory level. More computationally efficient methods, such as Density Functional based Tight Binding (DFTB), are under development to model the dynamics of these reactions (Aguirre et al., 2020; Carlson et al., 2020).

**FIGURE 4:**
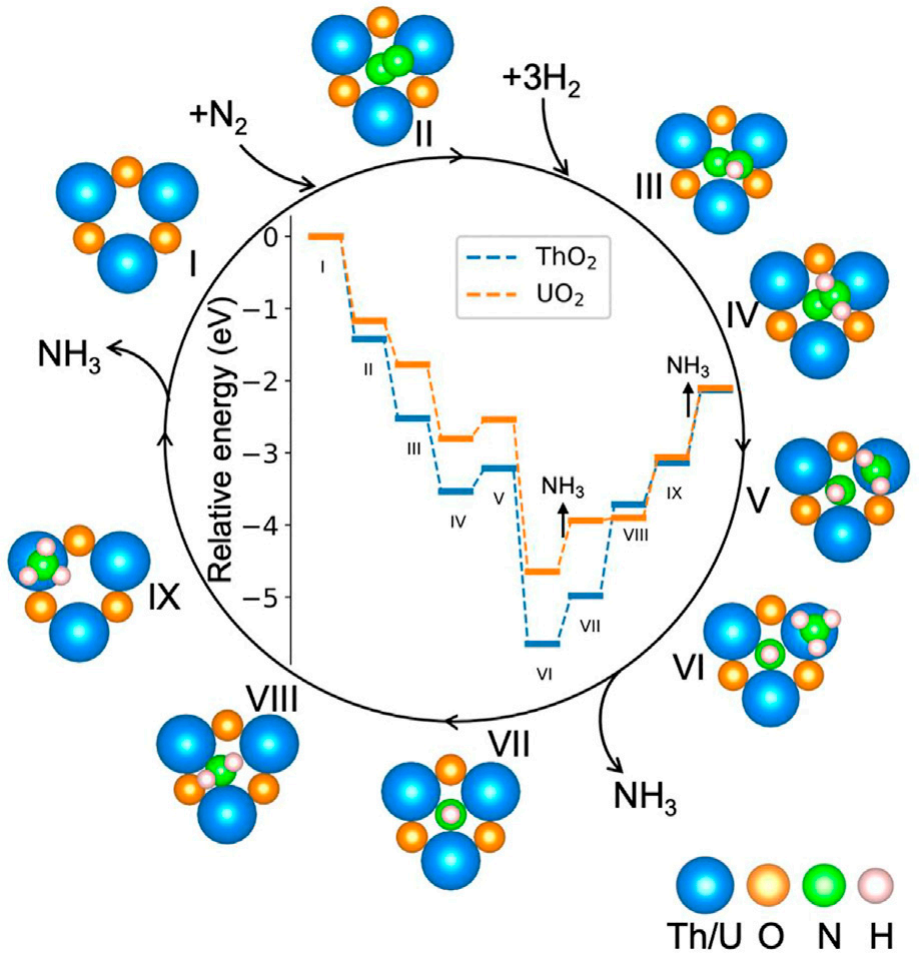
Potential energy diagram for the NH_3_ synthesis cycle on ThO_2_ and UO_2_ surfaces containing oxygen vacancies. The relative energy is defined by $\Delta E=E_{Total}-E_{sub}-E_{N_{2}}-3E_{H_{2}}$ , where $E_{Total}$ is the energy of the most stable configuration at each reaction step, as shown in the figure, $E_{sub}$ is the energy of the ThO_2_/UO_2_ surface containing the oxygen vacancy, and $E_{N_{2}}$ and $E_{H_{2}}$ are the energies of the N_2_ and H_2_ gas phase molecules, respectively.

**FIGURE 5:**
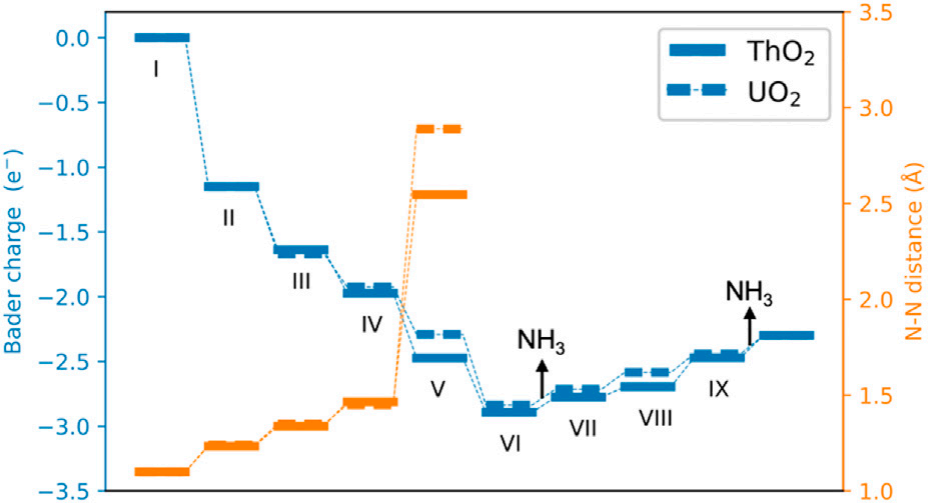
Bader charges and N−N distance during the catalytic cycle of NH_3_ formation. A negative value indicates that *NN obtains electrons from surfaces.

Figure 4 shows the calculated potential energy diagram for the NH_3_ synthesis cycle on ThO_2_ and UO_2_ surfaces containing oxygen vacancies. The most favorable computed pathway is N_2_ → *NN (II) → *NNH (III) → *NNH_2_ (IV) → *NHNH_2_ (V) → *NHNH_3_ (VI) → *NH + NH_3_ (VII) → *NH_2_ + NH_3_ (VIII) → *NH_3_ + NH_3_ (IX) → 2NH_3_. After the adsorption of N_2_ at the vacancy site, *NN is hydrogenated step-by-step through the associative pathway to form NH_3_. From the adsorption of N_2_ (I and II), to formation of the first *NH_3_ on the surface (VI), the reactions are exothermic except for the third hydrogenation process. The first (III) and second (IV) hydrogenations occur on the N atom with a higher position on the surface. Hydrogenation of the other N atom requires an additional 0.18 eV, as shown in Supplementary Figure S3A. The third (V) hydrogenation occurs on the other N atom, as seen from the energy comparison of the different structures in Supplementary Figure S3B. After the third (V) hydrogenation, the N−N bond lenght increases from 1.10 Å in the gas phase to 2.55 Å on ThO_2_ and 2.88 Å on UO_2_, see Figure 5 and Supplementary Table S1. This is a clear indication that *NN is fully dissociated during this step. The first *NH_3_ is formed on the surface after the fourth (VI) hydrogenation, as shown in Supplementary Figure S3C. The release of the first NH_3_ molecule requires ∼0.8 eV on both ThO_2_ and UO_2_ surfaces. The subsequent hydrogenations, from VII to XI, require increasing energies. The required energy from VI to IX is lower on the UO_2_ surface than on the ThO_2_ surface, indicating that NH_3_ synthesis is more efficient on the UO_2_ surface.

The Bader charges of nitrogen atoms during the catalytic reaction cycle are summarized in Figure 5. It is seen that after N_2_ adsorption, *NN possesses a Bader charge of 1.2 e^−^ due to charge transfer from the excess surface electrons to the hybrid π*−5*f* orbitals. *NN is further reduced step-by-step during hydrogenation, to give a Bader charge of 2.83 e^−^ in configuration VI. After the first NH_3_ release from the surface, the Bader charge of the remaining nitrogen atom on the surface becomes less negative, from 2.83 e^−^ to 2.30 e^−^ because it is gradually driven away from the vacancy site during hydrogenation from steps VII to IX, and the excess electrons return to the vacancy site in this part of the synthesis cycle. The surfaces containing oxygen vacancies recover to their initial states and are ready for the next catalytic cycle of NH_3_ synthesis.

## Discussion

The participation of excess electrons in the chemical reactions is critical to enable the proposed catalytic NH_3_ formation on ThO_2_ and UO_2_ surfaces. An illustration of the participation of excess electrons in the reactions is shown in Supplementary Figure S4. The excess electrons immediately transfer to N_2_ after adsorption at the vacancy site, weakening the N≡N bond. The hydrogenation of *NN further breaks the N−N bond and forms NH_3_. This associative *NN bond breaking process has been reported for NH_3_ synthesis using singly dispersed Fe_3_ or Rh_1_Co_3_ supported by Al_2_O_3_(010) or CoO(011) (Liu et al., 2018; Ma et al., 2018). After the release of NH_3_, the excess electrons return to the vacancy to enable the next catalytic cycle for NH_3_ synthesis. It should be noted that an oxygen vacancy has a formation energy of 6.95−2.43 eV on ThO_2_ and UO_2_ surfaces (Wang et al., 2019); however, oxygen vacancies can be artificially created by high energy collisions with the surfaces, for example, by artificial bombardment with Ar ions, as demonstrated in previous experiments (Senanayake et al., 2007a; Senanayake et al., 2007b). Compared with multicomponent heterogeneous catalysts composed of singly dispersed metal atoms or clusters on supporting substrates, the single-component ThO_2_ and UO_2_ surfaces containing oxygen vacancies are easier to experimentally synthesize and control. Since early actinides, in particular thorium and uranium, are relatively abundant in nature, ThO_2_ and UO_2_ surfaces are promising candidates for industrialization toward catalytic applications Table 1.

**TABLE 1:** Adsorption energies and activation barriers of H_2_ and N_2_ molecules on ThO_2_ and UO_2_ surfaces containing oxygen vacancies.

(In units of eV)		ThO_2_	UO_2_
Adsorption energy	H_2_	–1.68	–1.32
	N_2_	–1.42	–1.17
Activation barrier	H_2_	0.14	0.00
	N_2_	0.05	0.00

## Conclusion

In summary, we explored the prospect of actinide dioxide surfaces containing oxygen vacancies as catalysts for N_2_ molecule activation and NH_3_ synthesis using first-principle calculations. Our calculations reveal that N_2_ and H_2_ can be chemically adsorbed at surface oxygen vacancy sites on ThO_2_ and UO_2_, with a very smaller energy barrier. H_2_ can be directly dissociated at the vacancy site to form two H^−^. N_2_ can be activated by excess electron transfer to the molecule. Subsequently, NH_3_ can be formed by H^−^ migration on the surfaces through associative mechanisms. This is the first demonstration of the synthesis of NH_3_ by utilizing surface oxygen vacancy sites on actinide dioxide surfaces. Compared to single-atom catalysts, where precise control of the structure and the stability of the catalysts are extremely challenging (Speck et al., 2021), the proposed oxygen vacancies on ThO_2_ and UO_2_ surfaces can be fabricated by Ar ion sputtering, which are relatively easier to realize in experiments and, in this application, would serve as activators of the surface (Senanayake et al., 2007a; Senanayake et al., 2007b). These results demonstrate that 5*f*-element materials can serve as promising single-component catalysts for challenging chemical reactions that generally require multiple-component catalysts, thereby simplifying the synthetic process of catalysts.

## Data Availability

The original contributions presented in the study are included in the article/Supplementary Material; further inquiries can be directed to the corresponding authors.
