# Supplementary material for: N2-to-NH3 conversion by excess electrons trapped in point vacancies on 5f-element dioxide surfaces
Source: Front Chem. 2023 Jan 5;10:1051496. doi: 10.3389/fchem.2022.1051496 (PMC9849761; doi:10.3389/fchem.2022.1051496)
Supplement: Supplementary file 1 [file DataSheet1.docx]

# Supporting information for

**N_2_-to-NH_3_ conversion by excess electrons trapped in point vacancies on 5*f*-element dioxide surfaces**

Gaoxue Wang*, Enrique Batista*, and Ping Yang*

Los Alamos National Laboratory, Los Alamos, NM 87544

(November 1, 2022)

*Corresponding author:

[gaoxuew@lanl.gov](mailto:gaoxuew@lanl.gov)

[erb@lanl.gov](mailto:erb@lanl.gov)

[pyang@lanl.gov](mailto:pyang@lanl.gov)

Table S1. The *NN distance on ThO_2_ and UO_2_ surfaces, and the typical N≡N, N=N, and N-N bond lengths. (https://www.wiredchemist.com/chemistry/data/bond_energies_lengths.html)

| Distance of *NN on ThO_2_ and UO_2_ surfaces (Å) | | |
| --- | --- | --- |
|  | ThO_2_ | UO_2_ |
| N_2_ gas | 1.10 | 1.10 |
| *NN (II) | 1.23 | 1.24 |
| *NNH (III) | 1.34 | 1.35 |
| *NNH2 (IV) | 1.47 | 1.45 |
| *NHNH2 (V) | 2.54 | 2.89 |
| Typical bond length of N≡N, N=N and N-N (Å) | | |
| N≡N | 1.10 | |
| N=N | 1.25 | |
| N-N | 1.45 | |

Table S2. Bader charges of H* on the ThO_2_ and UO_2_ surfaces.

|  | ThO_2_ | UO_2_ |
| --- | --- | --- |
| H1* | -0.62 | -0.55 |
| H2* | -0.62 | -0.56 |
| H3* | -0.92 | -0.65 |
| H4* | +0.43 | +0.59 |


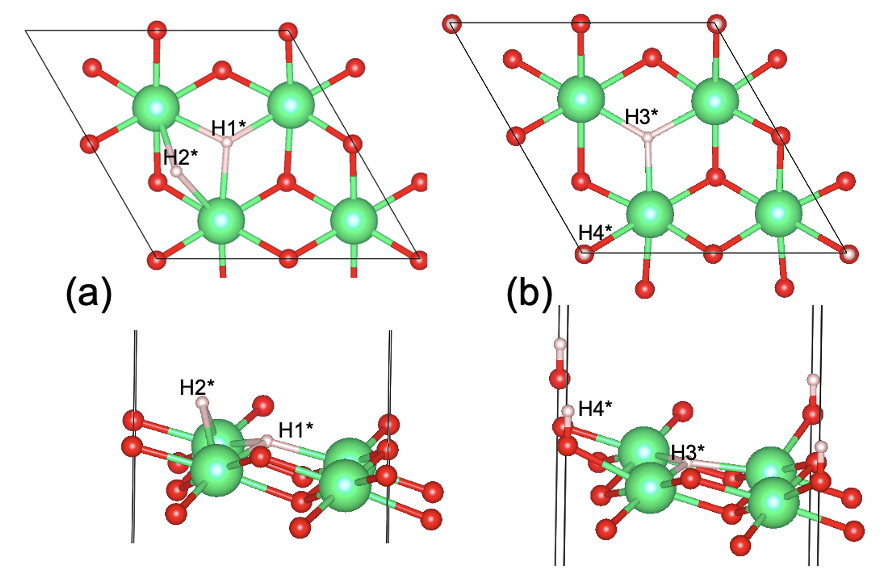


Figure S1. Structure and energy of H* on ThO_2_ surface. (a) Two H* on the surface, (b) one H* moves to a lattice O site forming OH* on the surface.


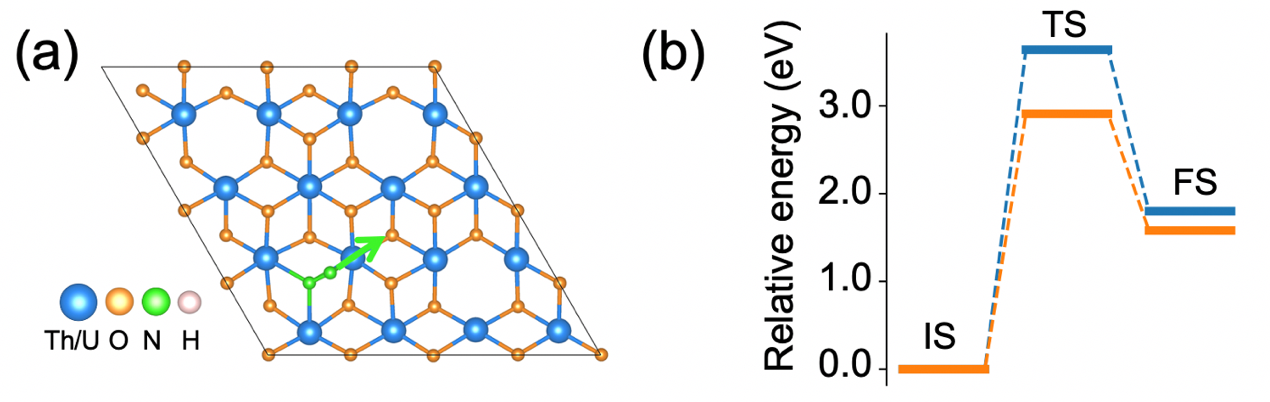


Figure S2. Direct dissociation of *NN at the vacancy site on ThO_2_ and UO_2_ surfaces. IS, TS, and FS in (b) represents the initial state, transiton state, and final state of N migration along the path in (a). The total energy of initial state is set to zero.


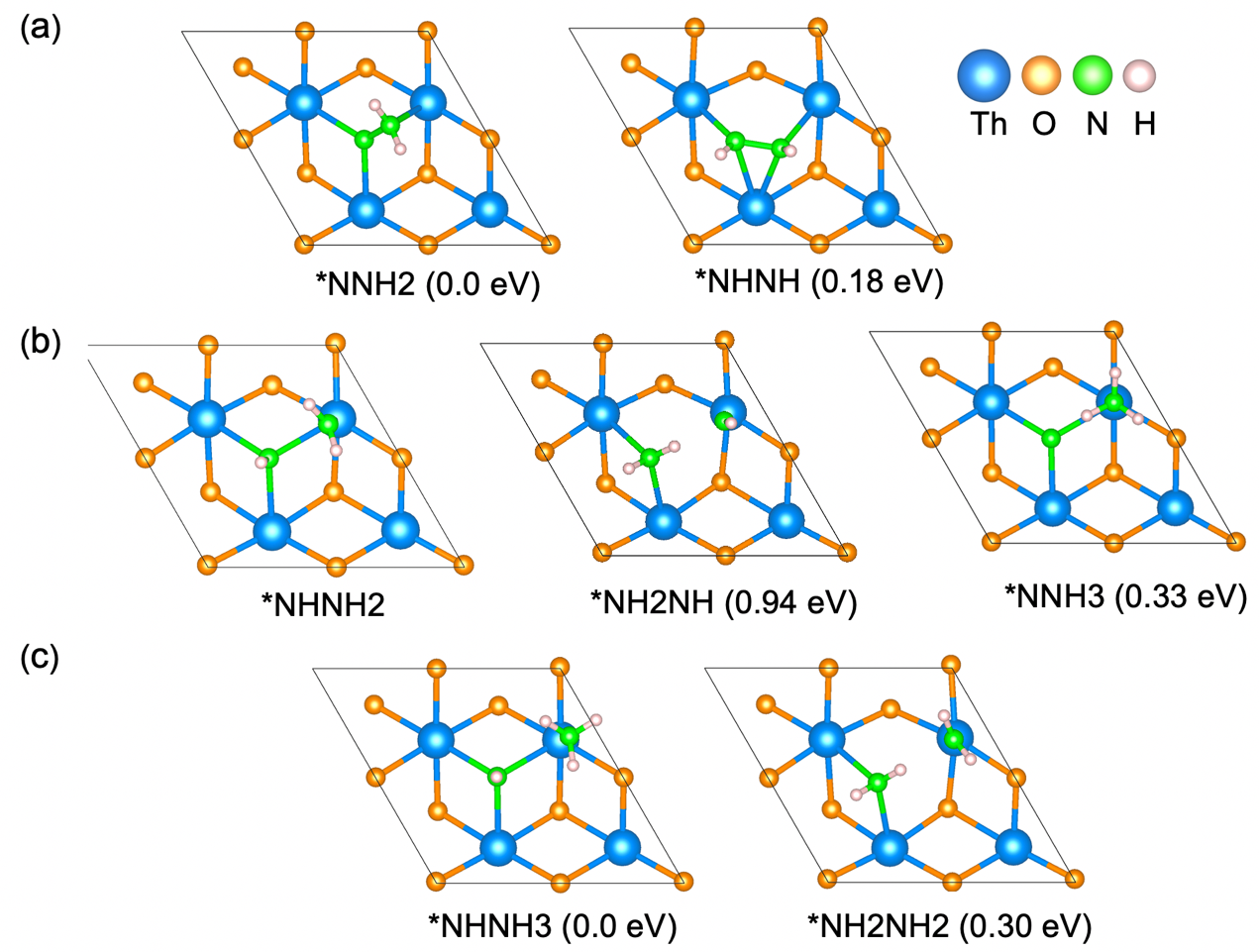
 Figure S3. Relative energies of different configurations compared to the most stable structure found in our calculations.


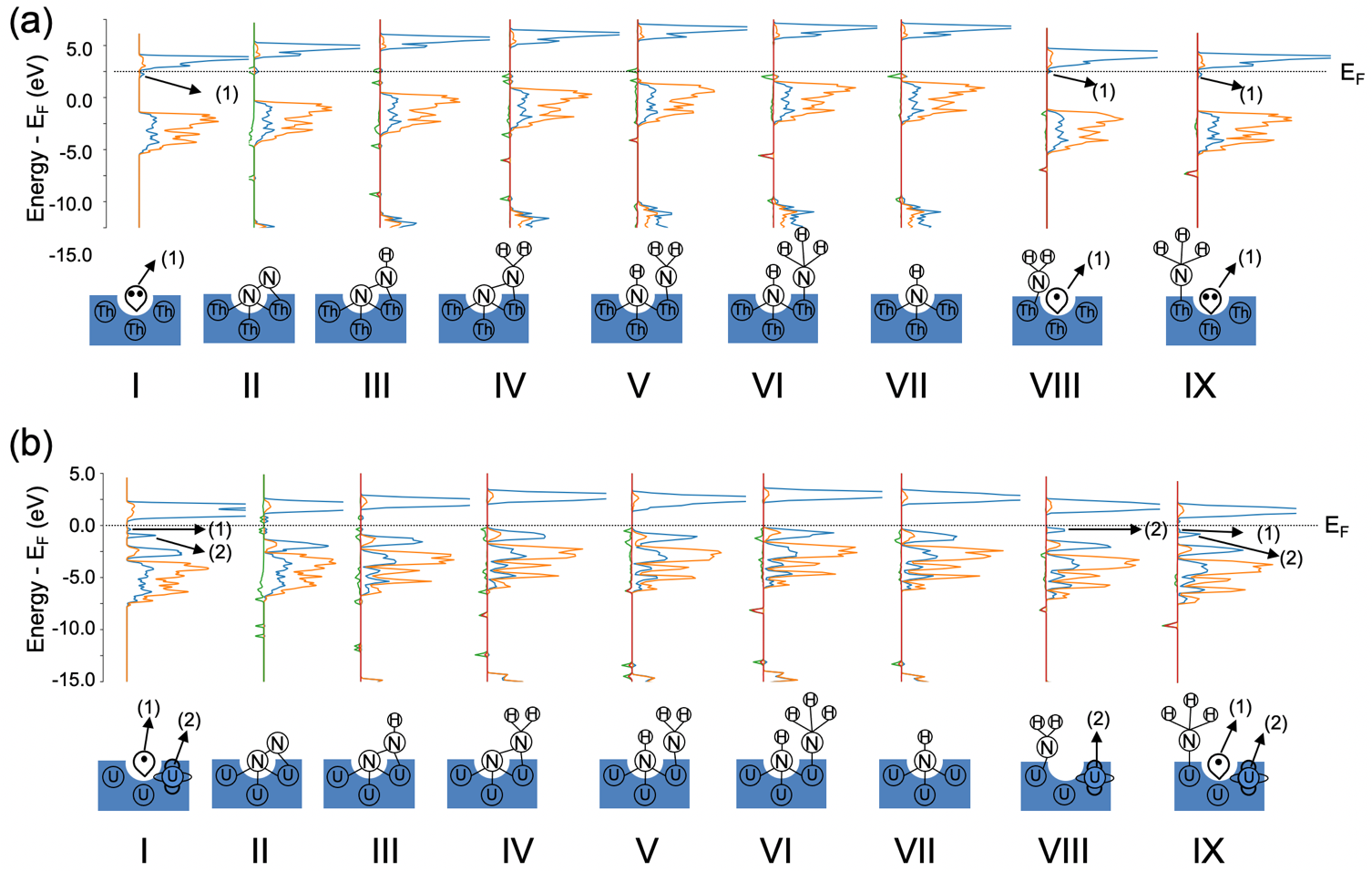
Figure S4. Density of states (DOS) and illustrations of the structures during the catalytic reaction cycle for the NH_3_ synthesis on ThO_2_ (a) and UO_2_ (b) surfaces wi nh oxygen vacancy. The labeled peaks in the DOS correspond to the excess electron
